# Dyadic Plasticity in Cardiomyocytes

**DOI:** 10.3389/fphys.2018.01773

**Published:** 2018-12-11

**Authors:** Peter P. Jones, Niall MacQuaide, William E. Louch

**Affiliations:** ^1^Department of Physiology, School of Biomedical Sciences, University of Otago, Dunedin, New Zealand; ^2^HeartOtago, University of Otago, Dunedin, New Zealand; ^3^Institute of Cardiovascular Sciences, University of Glasgow, Glasgow, United Kingdom; ^4^Clyde Biosciences, Glasgow, United Kingdom; ^5^Institute for Experimental Medical Research, Oslo University Hospital, University of Oslo, Oslo, Norway; ^6^KG Jebsen Center for Cardiac Research, University of Oslo, Oslo, Norway

**Keywords:** dyad, t-tubule, sarcoplasmic reticulum, calcium homeostasis, development, disease

## Abstract

Contraction of cardiomyocytes is dependent on sub-cellular structures called dyads, where invaginations of the surface membrane (t-tubules) form functional junctions with the sarcoplasmic reticulum (SR). Within each dyad, Ca^2+^ entry through t-tubular L-type Ca^2+^ channels (LTCCs) elicits Ca^2+^ release from closely apposed Ryanodine Receptors (RyRs) in the SR membrane. The efficiency of this process is dependent on the density and macroscale arrangement of dyads, but also on the nanoscale organization of LTCCs and RyRs within them. We presently review accumulating data demonstrating the remarkable plasticity of these structures. Dyads are known to form gradually during development, with progressive assembly of both t-tubules and junctional SR terminals, and precise trafficking of LTCCs and RyRs. While dyads can exhibit compensatory remodeling when required, dyadic degradation is believed to promote impaired contractility and arrythmogenesis in cardiac disease. Recent data indicate that this plasticity of dyadic structure/function is dependent on the regulatory proteins junctophilin-2, amphiphysin-2 (BIN1), and caveolin-3, which critically arrange dyadic membranes while stabilizing the position and activity of LTCCs and RyRs. Indeed, emerging evidence indicates that clustering of both channels enables “coupled gating”, implying that nanoscale localization and function are intimately linked, and may allow fine-tuning of LTCC-RyR crosstalk. We anticipate that improved understanding of dyadic plasticity will provide greater insight into the processes of cardiac compensation and decompensation, and new opportunities to target the basic mechanisms underlying heart disease.

## Introduction: Dyadic Organization at the Macroscale and Nanoscale

**FIGURE 1:**
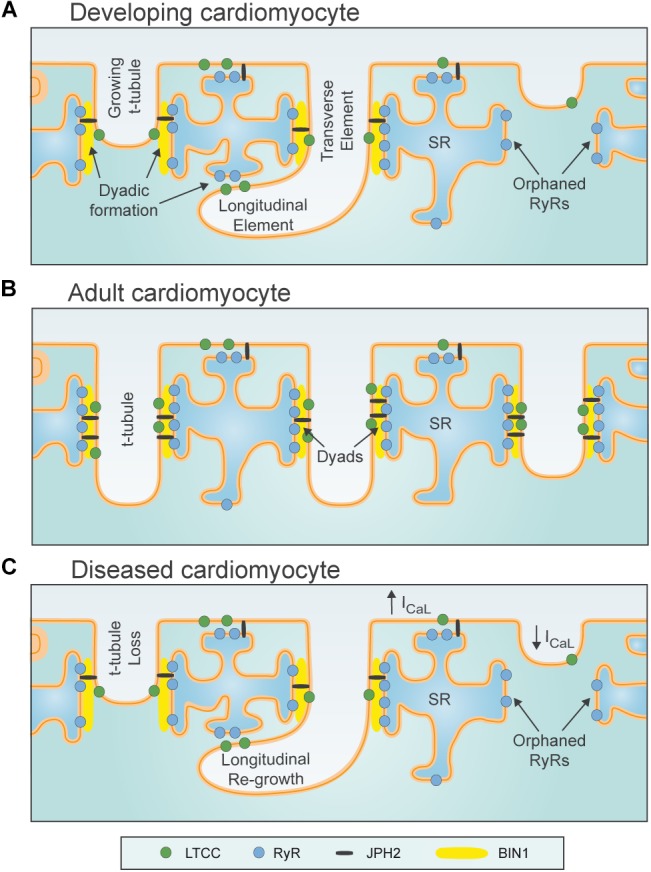
Plasticity of dyadic structure in ventricular cardiomyocytes. **(A)** Dyads form gradually in developing ventricular cardiomyocytes, as growing t-tubules extend the surface sarcolemma into the cell interior, initially in a largely longitudinal orientation. Rudimentary junctional SR terminals and contained ryanodine receptors (RyRs) are present in advance of t-tubule arrival. Formation of dyadic junctions between L-type Ca^2+^ channels (LTCCs) and RyRs requires the anchoring protein Junctophilin (JPH2), and the membrane sensing and bending protein BIN1. **(B)** Dyadic density increases toward adulthood, and assumes a predominantly transverse orientation. **(C)** During diseases such as heart failure, levels of JPH2 and BIN1 decline, and ventricular cardiomyocytes exhibit loss of t-tubules and SR. However, new dyads in the longitudinal orientation reappear, in resemblance to developing cells. T-tubule function also declines during heart failure, as L-type Ca^2+^ current (I_CaL_) is shifted to the surface sarcolemma.

In mammalian cardiac myocytes, contraction of the cell is elicited by a process known as excitation-contraction coupling. This process is initiated by electrical excitation during the cardiac action potential, which triggers the opening of voltage-gated L-type Ca^2+^ channels (LTCCs) present in both the surface membrane and within membrane invaginations called the transverse-axial tubule system (*t-tubules*). Ca^2+^ influx through LTCCs triggers release of Ca^2+^ from ryanodine receptors (RyRs) in the sarcoplasmic reticulum (SR), and contraction as this released Ca^2+^ binds to the myofilaments. Tight control of contractility thus requires efficient crosstalk between LTCCs and RyRs, which is afforded by close apposition of the sarcolemmal and SR membranes at junctions called *dyads* (Figure 1; Sun et al., 1995; Bers, 2001).

Adult ventricular cardiomyocytes generally have a well-organized network of dyads, with *transverse* elements predominantly arranged along z-lines at the ends of each sarcomere (Fawcett and McNutt, 1969; Brette and Orchard, 2003; Song et al., 2005; Louch et al., 2010). However, *longitudinal* or *axial* dyads are also present at the level of the A-band (between z-lines), where they are oriented along the long axis of the cell (Asghari et al., 2009; Swift et al., 2012; Pinali et al., 2013). Smaller mammalian species with high heart rates such as mice and rats exhibit high densities of dyads in both orientations, while a less dense dyadic network with fewer longitudinal tubules is present in ventricular cardiomyocytes from larger species (Brette and Orchard, 2003; Song et al., 2005; Louch et al., 2010). Atrial cardiomyocytes generally exhibit a lower dyadic density than ventricular cells, although dyadic organization varies across the atria (Lenaerts et al., 2009; Smyrnias et al., 2010; Richards et al., 2011; Dibb et al., 2013; Frisk et al., 2014; Glukhov et al., 2015; Gadeberg et al., 2016; Arora et al., 2017).

Dyadic density and organization have considerable functional implications. A high density of dyads ensures that Ca^2+^ release occurs evenly across the cell, resulting in a rapid and co-ordinated rise in intracellular Ca^2+^ concentration ([Ca^2+^]_i_) and rapid contraction. Of note, findings from a range of species indicate that RyR organization has greater regularity than the t-tubule network, resulting in the presence of “orphaned” or non-junctional RyRs along z-lines which do not have colocalized t-tubules (Louch et al., 2006; Song et al., 2006; Heinzel et al., 2008). Ca^2+^ release at these orphaned RyRs is delayed, as it is dependent on the diffusion of Ca^2+^ released from nearby RyRs. Thus, greater dyssynchrony and slowing of Ca^2+^ release is promoted by conditions which trigger loss of t-tubules (and dyads) including hyperosmotic shock (Brette et al., 2004, 2005), cell culture (Lipp et al., 1996; Louch et al., 2004), and diseases such as heart failure (Louch et al., 2006; Song et al., 2006; Heinzel et al., 2008).

Beyond macroscale considerations of the local presence or absence of dyads, the nanoscale arrangement of proteins *within* dyads is also of key importance. Recent studies employing electron microscopy (EM) and super-resolution imaging have indicated that dyads are not completely filled with RyRs, but often contain multiple, smaller RyR clusters (Baddeley et al., 2009; Hayashi et al., 2009; Jayasinghe et al., 2018; Kolstad et al., 2018). These considerations are essential for understanding Ca^2+^ sparks, the fundamental units of Ca^2+^ release in cardiomyocytes (Cheng et al., 1993). On the t-tubule side of the dyad, LTCCs are arranged opposite from RyR clusters. Interestingly, recent data suggest that neighboring LTCCs may be clustered and functionally paired (Dixon et al., 2012, 2015), in a manner somewhat reminiscent of groupings of neighboring RyRs (Marx et al., 2001; Sobie et al., 2006; Cabra et al., 2016). The precise mechanisms by which individual or grouped LTCCs may be anchored and apposed from RyRs is unclear, although the dyadic anchor junctophilin-2 (JPH2) has been shown to interact with both proteins (Jiang et al., 2016; Munro et al., 2016; Reynolds et al., 2016). More clear is the role that JPH2 plays in setting a consistent and remarkably narrow dyadic cleft (12–15 nm) (Sun et al., 1995; Takeshima et al., 2000) required for efficient LTCC-RyR crosstalk.

The above discussion has illustrated that considerable progress has been made into understanding dyadic organization and function in healthy adult cardiomyocytes. However, accumulating data indicate that these structures also exhibit remarkable plasticity. Indeed, dyads are known to form gradually during development (Ziman et al., 2010; Louch et al., 2015), and to exhibit compensatory remodeling when required (Kemi et al., 2011; Swift et al., 2012). In contrast, dyadic degradation is widely described during cardiac disease, where it is believed to contribute to impaired contractility and arrythmogenesis (Guo et al., 2013; Orchard et al., 2013; Manfra et al., 2017). In the remainder of this review, we will summarize how such plasticity of dyadic structure/function is attained, with focus on macroscale changes in t-tubule and SR structure, as well as nanoscale regulation of LTCCs and RyRs. Particular attention will be given to an emerging understanding of the drivers of dyadic plasticity, and their potential targeting for novel therapies.

### Macroscale Plasticity

#### T-Tubules During Development and Disease

**FIGURE 2:**
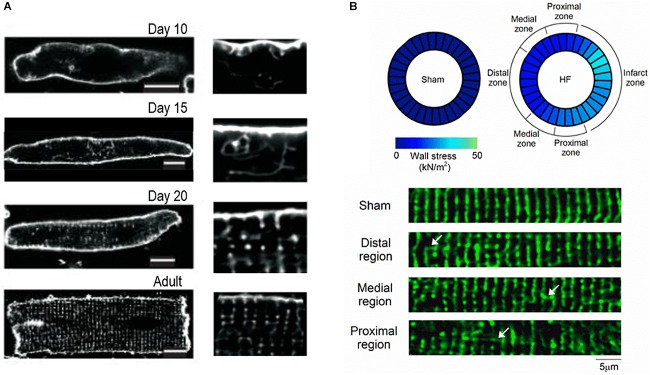
T-tubule plasticity during development and heart failure. **(A)** Confocal imaging of rat cardiomyocytes isolated at a range of post-natal time points reveals progressive t-tubule growth. T-tubules initially appear as a sparse network which is largely oriented in the longitudinal orientation, before the dense, predominantly transverse network is established in adulthood (whole cell images at left, with enlargements at right; adapted from Ziman et al. (2010); scale bar = 10 μm, copyright permission to reproduce the figure). **(B)** Typical t-tubule remodeling during heart failure exhibits a return to an immature phenotype, with loss of transverse elements and re-appearance of longitudinal elements (arrows). In a post-infarction rat model of heart failure, it was observed that remodeling is most marked proximal to the infarction scar, where *in vivo* wall stress is particularly elevated (adapted from Frisk et al. (2016), copyright permission to reproduce the figure). These data contribute to a growing understanding that high workload/wall stress signals t-tubule remodeling in this condition (reviewed in Ibrahim and Terracciano, 2013; Manfra et al., 2017).

In small rodents such as mice and rats, t-tubules form after birth, growing from the cell surface into the interior of the ventricular myocyte (Ziman et al., 2010; Louch et al., 2015; Mackova et al., 2017). Initially, this developing t-tubule network is rather disorganized in appearance, and oriented largely along the longitudinal axis of the cell (Figures 1A, 2A). With further maturation, t-tubule density increases and the network becomes predominantly transversely organized along z-lines; a process that continues until surprisingly late periods of adulthood (Ziman et al., 2010; Øyehaug et al., 2013; Louch et al., 2015; Mackova et al., 2017). Recent data indicate that sheep myocytes already exhibit t-tubules *in utero* (Munro and Soeller, 2016), supporting species-dependent differences in the time course of ventricular myocyte development. In either case, prenatal or postnatal t-tubule maturation coincides with expression of JPH2 (Ziman et al., 2010; Munro and Soeller, 2016), which critically forms dyads by anchoring MORN motifs in the t-tubular membrane to the junctional SR (Takeshima et al., 2000). Indeed, when JPH2 levels are reduced in mice, t-tubules either don’t appear or remain in an immature longitudinal configuration (Chen et al., 2013; Reynolds et al., 2013). Full knockout of JPH2 in mice results in *embryonic* mortality, consistent with a requirement of the protein to form dyads at the surface of the cell, in advance of t-tubule development (Takeshima et al., 2000; Franzini-Armstrong et al., 2005). The membrane sensing and bending protein Amphyphisin-2 (BIN1) is also reported to play a key role in t-tubule growth (Lee et al., 2002), and the intricate folding of the tubule inner membrane (Hong et al., 2014). Hong *et al* noted a particularly important role of a cardiac-specific isoform of BIN1 (isoform 13+17) which is capable of initiating t-tubule growth even in non-muscle cells (Hong et al., 2014). Assuming that BIN1 is essential for t-tubule development across a range of species, it is anticipated that this role is prominent at earlier stages in larger mammals which exhibit t-tubule development *in utero*.

**FIGURE 3:**
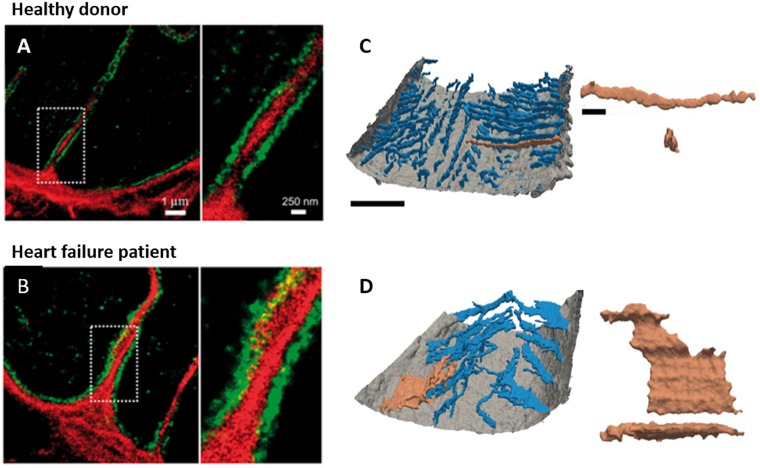
New insights into t-tubule remodeling during human heart failure. In comparison with ventricular tissue obtained from healthy donor hearts **(A**) tissue from heart failure patients undergoing transplant **(B)** revealed dilation of t-tubules associated with collagen deposition within the t-tubule lumen. Images were obtained with dSTORM super-resolution microscopy with staining for collagen VI (red) and dystrophin (green); enlargements of the indicated regions are shown at right (adapted from Crossman et al., 2017, copyright permission to reproduce the figure). Other recent work has indicated that in addition to t-tubule loss, cardiomyocytes in heart failure patients exhibit fusion of neighboring t-tubules into sheet-like structures (donor example in **C**, heart failure patient in **D)**. 3D reconstructions illustrate the surface sarcolemma (gray) and t-tubules (blue), with the indicated tubule enlarged at right (longitudinal and transverse views; left scale bar = 10 μm, right scale bar = 2 μm; adapted from Seidel et al., 2017a, copyright permission to reproduce the figure).

Evidence of t-tubule plasticity is further supported by examinations of cardiac disease. A large number of studies have reported remodeling of t-tubules in left ventricular cardiomyocytes during heart failure with an array of etiologies, spanning myocardial infarction (Louch et al., 2006; Swift et al., 2008; Lyon et al., 2009; Biesmans et al., 2011; Chen et al., 2012; Wagner et al., 2012; Øyehaug et al., 2013; Frisk et al., 2016; Sanchez-Alonso et al., 2016; Figure 2B), aortic stenosis (Wei et al., 2010; Ibrahim et al., 2013; Pinali et al., 2013), tachycardia (He et al., 2001; Balijepalli et al., 2003), hypertension (Song et al., 2006; Singh et al., 2017), chronic ischemia (Heinzel et al., 2008), and diabetes (Stølen et al., 2009; Ward and Crossman, 2014). Despite the range of species and disease models employed in these studies, there is general agreement that overall t-tubule density is reduced, and commonly accompanied by a re-emergence of longitudinally-oriented tubules (Figures 1C, 2B). More detailed analyses have revealed t-tubular swelling in failing myocytes (Wagner et al., 2012; Pinali et al., 2013, 2017; Crossman et al., 2017; Figures 3A,B) and the appearance of abnormal t-tubule “sheets” which may result from fusion of neighboring tubules (Seidel et al., 2017a; Figures 3C,D). Similar changes in t-tubule organization have been observed during right ventricular failure (Xie et al., 2012; Caldwell et al., 2014) and in the atria during heart failure (Dibb et al., 2009) and atrial fibrillation (Lenaerts et al., 2009), suggesting that t-tubular remodeling may be endemic to a variety of cardiac pathologies across the chambers of the heart.

Structural similarities between diseased and developing ventricular myocytes (Figure 1) have implied that pathological t-tubule remodeling may result from re-expression of fetal genes and/or suppression of adult genes in these conditions (Louch et al., 2015). Although the details of these mechanisms are still being elucidated, existing studies have already linked t-tubule remodeling in failing cells to declining expression of JPH2 (Minamisawa et al., 2004; Wei et al., 2010; Landstrom et al., 2011; Frisk et al., 2016), which is reminiscent of developing cells. An important role of JPH2 reduction in disease pathophysiology is supported by the observation that overexpression of this dyadic anchor protects against t-tubule degradation and heart failure development (Guo et al., 2014). Xu et al reported that JPH2 expression may be suppressed during disease by upregulation of microRNA-24 (miR-24), and showed that a miR-24 antagomir protected against changes in t-tubular architecture (Xu et al., 2012). Others have reported that JPH2 may be mislocalized in the failing heart, due to reorganization of microtubules necessary for its delivery to dyads (Zhang et al., 2014; Prins et al., 2016). JPH2 may also be degraded during heart failure by calpain cleavage. Guo et al. (2015) identified four distinct cut sites on JPH2 which resulted in functionally inactive fragments and disrupted dyadic junctions. Finally, recent data have suggested that JPH2’s functionality is dependent on its phosphorylation status. Quick et al. (2017) showed that JPH2 is phosphorylated by Striated Muscle Preferentially Expressed Protein Kinase (SPEG) and that this phosphorylation is reduced in heart failure, with knockout of SPEG also resulting in t-tubule disarray. These findings suggest that there may be several mechanisms which underlie loss of JPH2 expression and/or function during disease leading to pathologic disruption of t-tubule structure. Thus, JPH2 is an exciting potential candidate for future therapies aimed at preserving t-tubule integrity (Røe et al., 2015; Manfra et al., 2017).

BIN1 is another regulator of t-tubule structure which plays key roles in the developing and diseased heart. Indeed, just as BIN1 is believed crucial for the formation of t-tubules during development, so too has t-tubule loss and disarray during disease been linked to its downregulation (Lee et al., 2002; Caldwell et al., 2014; Hong et al., 2014). While the precise role of BIN1 in t-tubule growth and maintenance is unclear, it has been shown to group phosphoinositides allowing dynamin-2 polymerization; steps essential in tubulogenesis (Lee et al., 2002; Picas et al., 2014). Declining BIN1 levels in heart failure are reported to promote not only overall t-tubule loss (Caldwell et al., 2014), but also decreased t-tubule folding (Hong et al., 2014). Based on mathematical modeling studies, Hong et al. (2014) predicted that such loss of fine structure augments ion diffusion within the t-tubule, predisposing for cardiac arrhythmia. These authors have further proposed that continuous turnover of BIN1 from dyads in healthy patients maintains high levels in blood, explaining why decreased BIN1 plasma levels are linked to heart failure in patients and predict- future arrhythmia (Hong et al., 2012). These exciting data suggest that BIN1 may serve as both a biomarker and therapeutic target in heart failure patients.

While new molecular regulators such as JPH2 and BIN1 are emerging, recent work has also linked control of t-tubule structure to upstream mechanical signals. Experiments pioneered by the Terracciano group first indicated that t-tubule loss during heart failure may be directly triggered by the elevated ventricular workload in this condition. They observed that unloading failing hearts by heterotopic transplantation into healthy animals rescued t-tubule structure (Ibrahim et al., 2012a,b). Indeed, other strategies that unload the failing heart either pharmacologically (Chen et al., 2012; Xie et al., 2012; Huang et al., 2016) or via resynchronization therapy (Lichter et al., 2014), are similarly, protective. More recent work by our group indicated that elevated ventricular wall stress, which occurs in the dilated, thin-walled ventricle of the failing heart, may be the specific mechanical signal underlying t-tubule degradation (Frisk et al., 2016; Figure 2B).

How does mechanical overload lead to t-tubule degradation? While the precise mechanisms are unclear, it is important to consider that elevated workload and wall stress regulate not only cardiomyocyte remodeling but also promote changes in the extracellular matrix, including significant fibrosis. An exciting new study by Crossman et al., 2017; has shown striking localization of fibroblast filopodia and collagen within the t-tubular lumen in failing ventricular cardiomyocytes (Figures 3A,B). The authors suggested that such collagen deposition may directly drive t-tubular dilation in this condition, although it may also stiffen the t-tubule membrane, and impair normal mechanosignaling (McNary et al., 2012). Perhaps such changes mark a t-tubule for degradation (Louch and Nattel, 2017). In support of this view, regions of the failing heart with the most pronounced fibrosis, such as those proximal to an infarction, exhibit the most marked t-tubule loss (Frisk et al., 2016; Seidel et al., 2017b; Figure 2B). The Terracciano group has proposed that the stretch-sensitive protein titin cap (TCap) may play a key role in integrating these mechanical signals (Ibrahim and Terracciano, 2013; Ibrahim et al., 2013). With established binding proteins in the t-tubule membrane as well as partners in the cytoskeleton, TCap certainly appears to be well-positioned to serve such a function. Direct manipulation of the cytoskeleton has also been shown to regulate t-tubule structure, as cytoskeletal disruptors inhibit t-tubule loss during culture (Tian et al., 2012; Hodne et al., 2017). Recent data from the Song laboratory have further implicated protein kinase C activation as a critical determinant of cytoskeletal reorganization and t-tubule degradation (Guo et al., 2018). Taken together, these data raise the intriguing possibility that, by sensing local load, the t-tubule can regulate its own structure via signals transmitted from the extracellular matrix to the cytoskeleton.

Not all changes in t-tubule structure appear to be detrimental. At early stages of heart failure, longitudinal tubules appear before transverse elements have disappeared (Louch et al., 2006); changes which are suggested to be compensatory since additional Ca^2+^ influx at these sites supports the Ca^2+^ transient (Swift et al., 2012). However, a full understanding of the consequences of t-tubule dynamics for cardiomyocyte function requires detailed knowledge of SR structure, as well as the regulation of LTCCs and RyRs within these membranes. These topics are discussed in the following sections.

### Plasticity of SR Structure

**FIGURE 4:**
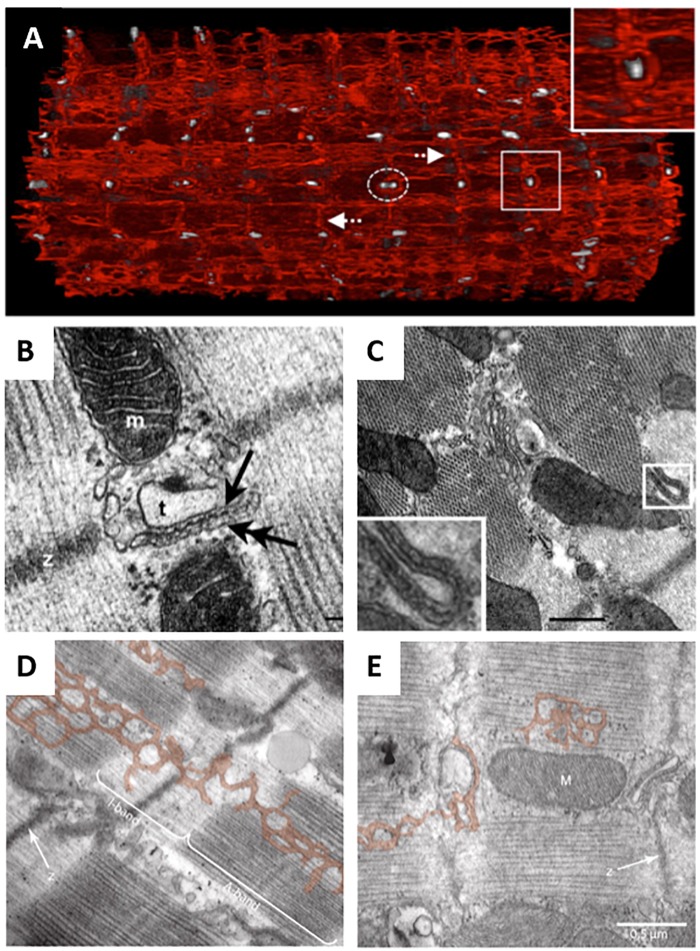
EM imaging of dyadic structure. **(A)** Block-face scanning EM performed on a sheep cardiomyocyte illustrates the complex, mesh-like nature of 3D SR structure (red), and it’s interrelationship with t-tubules (gray). Both longitudinal and perpendicular elements (arrows) are readily apparent, which converge to engulf t-tubules (enlarged region). Occasional twinning of t-tubules was observed with surrounding junctional SR (dashed elipsoid) (adapted from Pinali et al., 2013, copyright permission to reproduce the figure). Transmission EM imaging of transversely **(B)** and longitudinally-oriented dyads **(C)** revealed similar geometries, suggesting similar functionality of these structures (t, t-tubule; m, mitochondrion; double arrow, SR; scale bar in C = 100 nm). Ryanodine receptor heads are readily apparent (single arrow, adapted from Asghari et al., 2009, copyright permission to reproduce the figure). SR degradation during heart failure (Pinali et al., 2013) is suggested to be linked to reduction in SERCA levels, based on observations in the conditional SERCA knockout mouse (**D**, control cardiomyocyte; **E**, following SERCA knockout, with SR pseudo-colored; adapted from Swift et al., 2012, copyright permission was not required to reproduce the figure).

In comparison with t-tubule structure, SR structure has, in general, been less extensively studied. This is in part due to the fact that t-tubule structure is rather easily assessed by simple membrane staining and confocal microscopy; techniques which can be extended to 3D with relative ease. Direct staining and fluorescence imaging has not proven to effectively reveal SR structure, although junctional SR localization has been inferred from confocal immunostaining of RyRs (Bootman et al., 2006; Song et al., 2006; Swift et al., 2012) or calsequestrin (Terentyev et al., 2003). Greater detail has been provided by studies employing transmission EM, which revealed that the SR consists of a complex, branching network (Franzini-Armstrong, 1980). 3D structure has more recently been unveiled by serial block-face imaging with scanning EM, showing that the SR network is in fact contiguous between each t-tubule, is in regular contact with the surface membrane, and is variable between species (Pinali et al., 2013; Figure 4A). Importantly, the junctional SR forms dyads not only with the surface sarcolemma and along transversely-oriented t-tubules at z-lines, but also with longitudinally-oriented tubules within the A-band. EM studies have reported that these longitudinal dyads have similar dimensions to their transversely-oriented counterparts, suggesting that the two types of dyads may have similar functionality (Asghari et al., 2009; Pinali et al., 2013; Figures 4B,C).

Accumulating data suggest that, like t-tubules, SR structure is also malleable. Transmission EM imaging of developing hearts has shown that the junctional SR forms dyads with the surface sarcolemma from early stages of embryonic ventricular development (Franzini-Armstrong et al., 2005). However, rudimentary junctional SR terminals (cisternae) appear at internal sites along z-lines during the late embryonic stage (Korhonen et al., 2010), and remain present during the neonatal period prior to the arrival of growing t-tubules (Ziman et al., 2010). Thus, wavelike Ca^2+^ release patterns are observed, traveling from the cell membrane toward the cell interior, as Ca^2+^ release propagates between as yet “orphaned” RyRs. The subsequent arrival of t-tubules and formation of internal dyads synchronizes Ca^2+^ release across the cell, and has been linked to the presence of the dyadic anchoring protein JPH2, as described above (Chen et al., 2013; Reynolds et al., 2013).

Restructuring of the SR is also apparent during disease. Pinali et al. (2013) reported an overall loss of SR in sheep following tachypacing-induced heart failure, with local patchiness and disorder of SR observed near sites of abnormal mitochondrial clustering. The implications of such remodeling are unclear but imply that there may be disruption of Ca^2+^ fluxes within the network SR in diseased cells. Results from the SERCA knockout mouse suggest that SR degradation may be driven directly by SERCA loss during heart failure (Swift et al., 2012; Figures 4D,E). However, despite evidence of overall SR loss during heart failure, most report that the junctional SR and associated RyRs remain present along z-lines, at least in a rudimentary arrangement (Song et al., 2006; Swift et al., 2008; Pinali et al., 2013; Frisk et al., 2016). Thus, there is an increased presence of orphaned RyRs in diseased cells reminiscent of the developing heart (Louch et al., 2015). With fewer RyR clusters served by a t-tubule, Ca^2+^ release becomes desynchronized, as uncoupled CRUs are recruited by diffusion (Louch et al., 2004, 2006; Song et al., 2006; Heinzel et al., 2008). The resulting overall slowing and reduced amplitude of systolic Ca^2+^ release has been linked to reduced cardiac output in this condition (Bøkenes et al., 2008; Mørk et al., 2009; Guo et al., 2013; Røe et al., 2015). At sites where the junctional SR remains coupled to t-tubules, Wu et al. (2012) reported that there is shortening of the interface in failing cells as the SR terminals are shortened. This implies that there is less available space for LTCCs and RyRs within the dyad, which may impair triggering of Ca^2+^ release beyond effects associated with loss of t-tubules and reduced Ca^2+^ release synchrony. Importantly, while there may be some loss of SR structure along z-lines, there appears to be growth or at least specialization of SR within the A-band, allowing the formation of dyads with newly-grown longitudinal t-tubules (Song et al., 2006; Swift et al., 2012). It is hypothesized that these new dyads somewhat counterbalance those lost along z-lines, at least at early stages of disease, to help maintain Ca^2+^ release (Swift et al., 2012).

The above discussion has illustrated that t-tubule and SR structure exhibit considerable plasticity, which enables a malleable arrangement of dyads important for controlling the synchrony of Ca^2+^ homeostasis. In the following section we will describe emerging data indicating that there is also impressive plasticity of LTCCs and RyRs *within* dyads, consistent with nanoscale control of dyadic function.

## Nanoscale Plasticity

### Plasticity of LTCC Localization and Function

Whilst much is known of how the LTCC localizes to the triad in skeletal muscle, targeting of LTCCs to the cardiac dyad is more poorly understood. In skeletal muscle, LTCC positioning appears to be stabilized by both the presence of STAC3 (Nelson et al., 2013; Polster et al., 2016; Campiglio and Flucher, 2017) and direct physical interaction between the II-III loop of the channel and the skeletal muscle ryanodine receptor (RyR1) (Lu et al., 1994; El-Hayek et al., 1995). These interactions enable the formation of a distinctive tetrad arrangement, with 4 LTCCs apposed from 4 RyRs (Franzini-Armstrong et al., 1998; Takekura et al., 2004). However, cardiac muscle does not express STAC3 or a homologous protein nor does the cardiac isoform of the LTCC have a physical interaction with RyR (c.f. Dulhunty et al., 2005), leading to the question of how the channel is targeted.

At both the surface of cardiomyocytes and within t-tubules, LTCCs are found resident within caveolae. The bulk of channel delivery to these caveloae appears to be dependent on BIN1, which couples the channels to microtubules (Hong et al., 2010). However, the actin filament cytoskeleton is also proposed to play a role in LTCC trafficking, at least in neurons and recombinant cell lines (Hall et al., 2013; Ghosh et al., 2018). Once delivered, LTCCs are maintained within the dyad via links between the caveolae and cytoskeleton (Head et al., 2006; Balijepalli and Kamp, 2008). Interestingly, BIN1 may also help maintain LTCC positioning, as BIN1-induced microfolds within the t-tubule membrane are suggested to prevent lateral movement of the caveolae (Basheer and Shaw, 2016). Further evidence of the importance of BIN1 for LTCC maintenance is provided by Hong et al. (2012), who showed that in human heart failure there is no change in LTCC expression but a significant reduction in dyadic channels. Such loss of dyadic LTCCs correlates well with a reduction in the expression of BIN1, both in failing patients (Hong et al., 2012) and sheep (Caldwell et al., 2014). Finally, evidence from skeletal muscle indicates that JPH2 may also play a role in maintaining LTCCs as part of a dyadic protein complex (Golini et al., 2011), suggesting that JPH2 reduction during heart failure could have complex effects on both LTCC localization and overall dyadic structure. The mechanism by which un-anchored LTCCs are degraded is presently unclear, although indirect evidence showing that dynasore increases surface LTCC expression indicates that channel internalization may occur via dynamin-dependent endocytosis (Hong et al., 2010).

While the above discussion has highlighted an important role of caveolae in clustering LTCCs within dyads, accumulating data suggest that these structural arrangements also critically regulate channel function. Recent studies have shown that Caveolin-3 (Cav-3), which is known to play an important role in the formation of caveolae and t-tubules (Parton et al., 1997), interacts with both LTCCs and protein-kinase A (PKA) to enable PKA-mediated phosphorylation of the channel and augmentation of L-type current (Kamp and Hell, 2000). Indeed, using peptide mimics of the scaffolding domain of Cav-3 to disrupt this interaction, Bryant et al. (2014) observed reduction in both basal L-type function and its response to β-adrenergic stimulation. More recently the same group has gone on to show that in heart failure the loss of t-tubular Cav-3 reduces t-tubular L-type current, despite the continued presence of L-type channels (Bryant et al., 2015, 2018; Figure 1C). Thus, while loss of t-tubules promotes dyssynchronous release in failing ventricular cardiomyocytes, as discussed above, it seems that there is also an important Cav-3-dependent loss of functionality in remaining tubules which further compromises Ca^2+^ release in this condition. Interestingly, the loss of t-tubular L-type current appears to be paralleled by increased current on the cell surface (Bryant et al., 2015; Sanchez-Alonso et al., 2016), likely explaining why many groups have reported unchanged overall current density in heart failure (Gomez et al., 1997; Benitah et al., 2002; Kamp and He, 2002; Mørk et al., 2009). The Gorelik group has proposed that increased LTCC activity on the surface sarcolemma results from physical movement of channels out of dyads and onto the membrane crests present between Z-grooves (Sanchez-Alonso et al., 2016). They further suggest that the delocalization-induced increase in channel activity promotes instability of myocyte membrane potential and a concomitant increase in arrhythmias often associated with heart failure (Sanchez-Alonso et al., 2016). These findings raise the possibility that nanoscale LTCC localization might be therapeutically targeted in disease.

Finally, exciting recent data indicate that LTCC activity is also critically regulated by the physical clustering of the channels themselves. Using single channel electrophysiology and optical channel recordings in neonatal myocytes, Navedo et al. (2010) found that clustered LTCCs open together more frequently than stochastic opening would predict. The same group went on to show in ventricular myocytes that this functional coupling of LTCCs occurs through the physical interaction of their C-terminal tails (Dixon et al., 2012, 2015). It is likely that such coupling ensures that Ca^2+^ influx is rapid and large enough to drive efficient RyR opening during Ca^2+^-induced Ca^2+^ release. In light of findings described above, it seems plausible that loss of t-tubular L-type current during heart failure (Bryant et al., 2015; Sanchez-Alonso et al., 2016) may, at least in part, result from loss of channel clustering due to downregulation of Cav-3, BIN1, and/or JPH2.

### Plasticity of RyR Organization

#### Inter-Cluster RyR Dynamics

**FIGURE 5:**
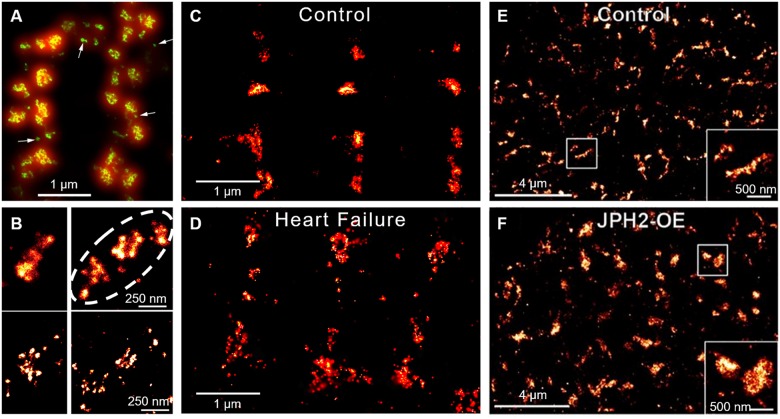
Super-resolution imaging of RyR clusters and plasticity. **(A)** RyRs on the cell surface of ventricular cardiomyocytes form clusters primarily along either side of the z-lines (double rows of RyRs). The limited resolution of conventional confocal imaging (shown red) is markedly improved using the dSTORM technique (green), which reveals the range of size and morphologies of RyR clusters. dSTORM also allows the visualization of smaller clusters and single RyRs (arrows, from Baddeley et al., 2009, copyright permission was not required to reproduce the figure). **(B)** Neighboring clusters can form superclusters or Ca^2+^ release units (CRUs) (dotted line; upper panel). DNA-PAINT reveals that RyRs within clusters are found in various orientations and groupings (lower panel, from Jayasinghe et al., 2018, copyright permission to reproduce the figure). **(C,D)** During heart failure, RyR clusters are broken apart, resulting in dispersed CRUs (from Kolstad et al., 2018, copyright permission was not required to reproduce the figure). **(E,F)** In contrast, RyR cluster size is increased in response to JPH2 overexpression (from Munro et al., 2016, copyright permission to reproduce the figure).

**FIGURE 6:**
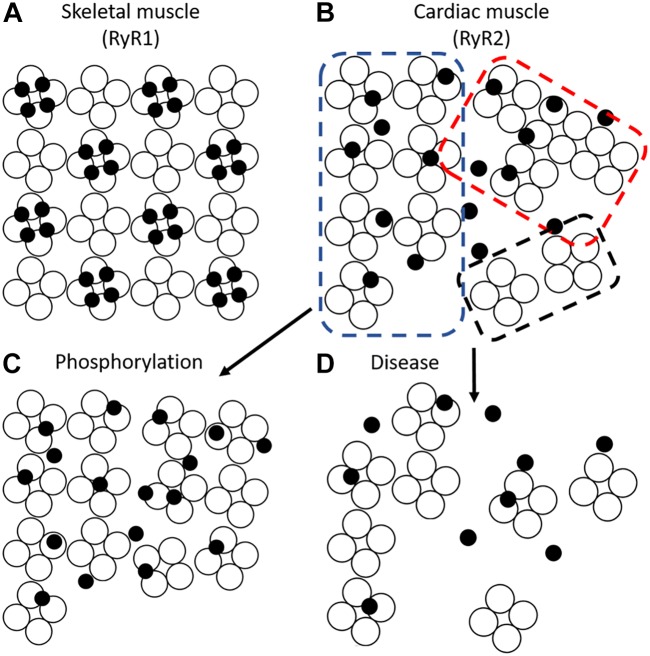
RyR cluster conformations. **(A)** Skeletal muscle RyRs (RyR1) form a regular crystalline array with 4 four LTCCs (filled circles) apposing alternate RyRs (open circles) to form tetrads. **(B)** Cardiac muscle RyRs (RyR2) interact in several conformations including crystalline array (blue dotted box), side-by-side (red dotted box), and disordered arrangements (black dotted box). Different stimuli modify the predominant conformation; phosphorylation promotes a crystalline array which may enhance the apposition of LTCCs and RyRs **(C)**. Pathological conditions are reported to promote RyR cluster dispersion (Louch et al., 2013; Macquaide et al., 2015; Kolstad et al., 2018) and mislocalize t-tubules, thus, decreasing the coupling between LTCCs and RyRs **(D)**.

While it was traditionally believed that dyads in ventricular myocytes are uniformly packed with RyRs, more recent super-resolution microscopy studies have indicated that dyads are in fact composed of sub-clusters (Baddeley et al., 2009; Hayashi et al., 2009; Jayasinghe et al., 2018; Kolstad et al., 2018; Figures 5A,B). Neighboring RyR clusters with sufficiently short distances between them (<100 nm in Sobie et al., 2006; <150 nm in Macquaide et al., 2015) are suggested to concertively generate Ca^2+^ sparks, as released Ca^2+^ can effectively jump from one cluster to the next. These functional groupings have thus been termed “superclusters” or Ca^2+^ release units (CRUs) (Baddeley et al., 2009), and may provide opportunities to fine tune spark dynamics (Walker et al., 2014). EM data suggest that RyR clusters, and presumably CRUs, are assembled gradually during development, first at the cell surface and then within the cell interior (Franzini-Armstrong et al., 2005). This process may be reversed during disease, as emerging data from our laboratories indicate that RyR clusters are broken apart (Macquaide et al., 2015; Kolstad et al., 2018; Shen et al., 2018; Figures 5C,D, 6). We have specifically linked dispersion of RyR clusters and CRUs during post-infarction heart failure to slowing of Ca^2+^ spark kinetics, due to the time lag inherent as multiple clusters are sequentially activated (Louch et al., 2013; Kolstad et al., 2018; Shen et al., 2018). Slowing of Ca^2+^ sparks in these cells was additionally linked to de-synchronization and slowing of the overall Ca^2+^ transient (Louch et al., 2013; Kolstad et al., 2018). We observed similar fragmentation of RyR clusters and slowing of Ca^2+^ spark kinetics during atrial fibrillation (Macquaide et al., 2015). An associated increased fraction of RyRs located between Z-lines was further predicted to augment propagation of pro-arrhythmic Ca^2+^ waves. Thus, accumulating data indicate that RyR clusters exhibit marked plasticity of their organization and function, and that this malleability has important implications for pathophysiology.

What is the timescale of RyR cluster dynamics? Some insight has been provided by a recent study employing direct visualization of RyR clusters using GFP-labeled RyR in live cells (Hiess et al., 2018). The authors observed that clusters close to the periphery of cells can undergo movement either laterally along the cell surface or down toward the interior. This movement altered cluster size as both cluster fusion and fission were observed. Remarkably, over a 12 min period movements of up to 1 μm occurred, which was sufficient for clusters to traverse half a sarcomere. Importantly, not all neighboring clusters repositioned, suggesting that cluster rearrangement was not an artifact due to SR movement (Hiess et al., 2018).

Importantly, inter-cluster dynamics appear to be controlled by external stimuli. Stimulatory conditions such as high Ca^2+^ were found to promote movement whereas conditions known to suppress RyR function such as low Ca^2+^ and tetracaine retarded their movement (Hiess *et al.*, 2018). This raises the possibility that hyperactivity of the RyR, a hallmark of diseased cardiomyocytes (Bers, 2014; Dries et al., 2018), directly promotes its migration. However, it has been suggested that RyR localization is also dependent on associated dyadic proteins. BIN1 is reported to traffic RyRs to the t-tubule during β-adrenergic stimulation (Fu et al., 2016), with a time scale that appears to be consistent with the rate of RyR cluster movements reported by Hiess et al. (2018). JPH2 may also play a role in RyR arrangement, as overexpression of the protein was observed to augment RyR cluster size (Munro et al., 2016; Figures 5E,F). Interestingly, in these larger clusters JPH2 expression appears to stabilize RyR activity (van Oort et al., 2011; Beavers et al., 2013; Reynolds et al., 2016). These observations suggest that both dispersion of RyRs and increased channel activity during heart failure and atrial fibrillation could be linked to downregulation of BIN1 and JPH2 in these conditions.

#### Intra-Cluster RyR Dynamics

Although super-resolution imaging techniques such as dSTORM have yielded a wealth of information about RyR cluster size and distribution, their ability to examine intra-cluster architecture is limited. A recent breakthrough by the Soeller group employed the DNA-paint technique, which yielded <10 nm resolution (Jayasinghe et al., 2018). These measurements allowed the first visualization of single channels using optical means. Jayasinghe et al found that most clusters contained largely disordered channels with a relatively low packing density (Jayasinghe et al., 2018). This finding disagrees with historical assumptions of ventricular cardiomyocyte RyR packing based on EM descriptions of skeletal muscle, which showed a crystalline checkerboard arrangement of RyR1 (Ferguson et al., 1984; Franzini-Armstrong et al., 1999). More recent EM studies have suggested a *somewhat* structured arrangement with cardiac RyRs predominantly organized in a checkerboard pattern (∼50% of channels) but with many channels present in side-by-side or disordered arrangements (Asghari et al., 2014; Figure 6). Strikingly, as with clusters themselves, the orientation of channels within a cluster appears highly plastic. For example, exposure of cells to high Mg^2+^ conditions was observed to drive clusters into a side-by-side orientation, whereas low Mg^2+^ favored checkerboard. Most dramatically, however, was the finding that phosphorylated channels appeared almost exclusively in a checkerboard orientation (Asghari et al., 2014) (Figure 6). These findings suggest that the crystalline array favors greater RyR activity. More recently, the same group has refined their model with data indicating that it is phosphorylation of RyR at S2030 and S2814 that drives the change in cluster orientation (Asghari et al., 2017). Whether other established post-translational modifications of RyR such as oxidation (Waddell et al., 2016) or agonists such as caffeine (Jones et al., 2008) also result in similar changes in orientation remains unknown.

Given that the clamp region of RyR is thought to enable assembly of the crystalline array, and that this region undergoes substantial movement during the transition of a channel to an open state, it is plausible that re-arrangement into the checkerboard pattern enhances coupled gating of channels (Cabra et al., 2016). Coupled gating has been previously proposed based on recordings of RyRs in bilayers, and implies that neighboring RyRs within a cluster exhibit synchronization of their opening and closing (Marx et al., 2001; Sobie et al., 2006). This mechanism has been suggested to be facilitated by FK506-binding protein (FKBP12.6), however, a role for this accessory protein in controlling RyR function remains controversial (Marx et al., 2000, 2001; Zhang et al., 2016; Gonano and Jones, 2017). Regardless of mechanism, an increase in coupled gating during transition to the checkerboard arrangement appears essential, as Walker et al. (2015) suggest that the increased intra-channel spacing in this configuration would otherwise *reduce* the likelihood of spark occurrence. However, the concept of coupled gating remains contentious as direct evidence linking cluster orientation and single channel activity is lacking (Williams et al., 2018).

In support of the work of the Moore group (Asghari et al., 2014), Samso and co-workers employed an alternative approach to examine RyR cluster formation, namely self-association in solution (Cabra et al., 2016). They found that akin to the patterns observed in cardiac muscle, *in vitro* RyRs adopt two major arrangements, namely side-by-side (further classified as center to side and adjoining) and checkerboard (further classified as oblique and center-to-corner). At low Ca^2+^ concentrations the channels were found to be relatively evenly distributed between the two conformations but, consistent with Asghari et al., stimulatory conditions (high Ca^2+^) favored an oblique and center-to-corner conformation, the basis of the crystalline array (Cabra et al., 2016). Given that stimulatory conditions such as high Ca^2+^ levels or phosphorylation appear to also increase both intra- and inter-cluster mobility, it would be of interest to examine the relative mobility of checkerboard and side-by-side orientated clusters in future studies.

## Plasticity of LTCC-RyR Coupling

The preceding sections have illustrated that there is remarkable plasticity of not only the membranes of the dyad, but also their contained LTCCs and RyRs. The malleable activity of these proteins is mediated through direct changes to single channel function but also through the coordinated movement and organization of the channels which provides another layer of control. However, our knowledge of these phenomena is largely limited to LTCCs and RyRs individually; whether plasticity of the two proteins is coordinated, or indeed if the molecular stimuli are shared, remains less clear. The dynamic clustering observed in response to β-adrenergic stimulation of both channels might not only increase each channel’s activity but also hints at more effective coupling through optimization of LTCC and RyR apposition. Perhaps the phosphorylation-induced transition of RyR to a checkerboard conformation might, similarly, position LTCCs into an arrangement more similar to that found in skeletal muscle. Similarly, it is plausible that the loss of dyadic channels in disease not only reduces their own function but, due to an altered nano-structural arrangement, also results in a further reduction in Ca^2+^ coupling between the remaining channels. Since molecular players such as JPH2, BIN1, and Cav-3 control the localization of both LTCCs and RyRs, perhaps downregulation of these regulators critically reduces channel alignment in diseased cardiomyocytes. More indirect effects might also be important, as changes to the t-tubule architecture such as dilation or swelling (Wagner et al., 2012; Pinali et al., 2013, 2017; Crossman et al., 2017) could also “misalign” or otherwise disrupt the functional coupling between the channels. This form of remodeling has been hypothesized for more than 20 years, based on an observed reduction in the “gain” of Ca^2+^-induced Ca^2+^ release (Gomez et al., 1997; Litwin et al., 2000). However, it is only now with the host of recent technological advances, that we might examine this concept experimentally, by combining gain measurements with live-cell imaging of LTCCs and RyRs.

## Conclusion

This review has presented a growing body of evidence illustrating that the concept of a static dyad severely underestimates the complexity of the structure. Rather, it is now fully apparent that there is remarkable plasticity of both t-tubule and SR structure, which enables dynamic dyad formation and degradation. Furthermore, it appears that within these structures there is likely continual regulation of both the positioning and activity of LTCCs and RyRs. This plasticity is postulated to augment dyadic Ca^2+^ cycling when required, but also to underlie impaired Ca^2+^ cycling during disease. Thus, greater understanding of dyadic plasticity holds considerable therapeutic potential.

## Author Contributions

All authors contributed to the conceptualization, drafting, and editing of the manuscript.

## Conflict of Interest Statement

NM was employed by Clyde Biosciences (Glasgow, United Kingdom). The remaining authors declare that the research was conducted in the absence of any commercial or financial relationships that could be construed as a potential conflict of interest.
